# Supplementary material for: Engines of change: Transposable element mutation rates are high and variable within Daphnia magna
Source: PLoS Genet. 2021 Nov 1;17(11):e1009827. doi: 10.1371/journal.pgen.1009827 (PMC8594854; doi:10.1371/journal.pgen.1009827)
Supplement: S1 Text — (DOCX) [file pgen.1009827.s001.docx]

**S1 Text: Supplemental Methods**

*DNA Extraction and Sequencing*

DNA was extracted using the Zymo Quick-DNA Universal Solid Tissue Prep Kit (No. D4069) following the manufacturer’s protocol (DNA from a few samples was also extracted with the Qiagen DNeasy Blood and Tissue Kit, No. 69504). DNA quality was assessed by electrophoresis on 3% agarose gels and DNA concentration was determined by dsDNA HS Qubit Assay (Molecular Probes by Life Technologies, No. Q32851). The Center for Genome Research and Biocomputing at Oregon State University generated 94 Wafergen DNA 150 bp paired-end libraries using the Biosystems Apollo 324 NGS library prep system. Quality was assessed using a Bioanalyzer 2100 (Agilent Technologies, No. G2939BA) and libraries were pooled based on qPCR concentrations across 16 lanes (2 runs). Libraries were sequenced on an Illumina HiSeq 3000 (150 bp PE reads) with an average insert size of ~380bp.

*Genome Assembly*

To reduce reference bias, we searched for TEs using reference-guided assemblies for each of the 9 genotypes (using the WGS from each SC). Reads from each sample were processed by trimming adaptor sequences (k=23, ktrim=r, mink=4, hdist=1, tpe, tbo), merging overlapping pairs (vstrict=t), and quality filtering (qtrim = rl, trimq=20, minlen=50) with BBTools [1]. First pass reference-guided *de novo* assemblies were performed with SPAdes (using the *trusted-contigs* option; [2]). The *D. magna* reference used to guide the assembly was provided by Peter Fields and Dieter Ebert (accession: LRGB00000000). To remove haplotigs potentially derived from assembling heterozygous regions into alternate alleles, we collapsed the filtered assemblies with *redundans* by implementing information from paired-end reads, merged reads, and the *D. magna* reference genome for scaffolding [3]. We then mapped the processed reads of each SC onto the collapsed assemblies with BWA-MEM v0.7.17 [4]. Contigs were removed if they possessed an average depth of coverage < 5 or were shorter than 5 kb.

*Characterizing TE Abundance and Diversity, Insertion Site Polymorphism and Mean Pairwise Divergence*

For each assembly, we calculated the proportional abundance of each TE class or family by dividing the sum of their lengths by the total length of the assembly. We also used a read-mapping approach to calculate the TE fraction of the genome. For each assembly, we clustered elements in the TE library if they exhibited at least 98% nucleotide identity over their full length to a longer sequence in the library with the program cd-hit-est v4.8.1 [5], yielding a non-redundant TE library containing full and partial TE copies. Elements with matches to sequences in the consensus library shorter than 150 bp, the average sequence read length, and identified as “Unknown” were excluded from this non-redundant TE library. Processed reads were then mapped to the non-redundant TE library with BWA-MEM v0.7.17 [4]. The number of bp in the genome covered by a particular TE copy was determined by summing the lengths of reads mapped to each TE sequence and normalizing to the median coverage of single-copy regions for each sample. The lengths of all constituent TE copies were summed to estimate the total number of bp in each genome and divided by the length of the assembly to obtain percent occupancy.

To estimate the insertion site polymorphism (i.e., the number of TEs at a particular site in a genome shared by multiple genotypes) we used TEFLoN v0.4 [6]. TEFLoN allows users to determine the presence/absence patterns of TEs among lineages, either among natural isolates (i.e., our 9 starting genotypes) or among MA lines (i.e., the MA lines derived from each starting genotype). ‘Presence’ reads are soft-clipped at a TE insertion site, or have one end of a pair mapped to a TE copy while the other maps to a unique position in the pseudo-reference, while ‘absence’ reads span an insertion site in the pseudo-reference. Pseudo-references are created by removing all matches to TEs in the consensus library identified with RepeatMasker v4.1.0 [7] that are >150 bp. Non-mergeable, quality-filtered paired end reads are mapped to pseudo-references for corresponding SCs with BWA-MEM v0.7.17 [4], with soft-clipping for supplementary alignments (-Y). As a result, TEFLoN reveals the presence/absence status at each site (TE insertion polymorphism [TIP]), although it does not return the specific internal sequence that occupies/does not occupy scored sites.

We specified an empirically determined insert size standard deviation of 150 bp, and removed TEs identified within 5 bp of the end of any scaffold. To identify TIPs, we mapped reads from each of the 9 SCs to the pseudo-reference of a focal SC, and repeated this using all 9 SCs as the focal. We required that all occupied/unoccupied sites were supported by ≥ 20 presence + absence reads with mapping quality ≥ 30. A TE copy was considered present in a line if the frequency of the presence allele was ≥ 0.05 (altering this threshold did not substantially alter the distribution of TIPs identified using each reference). Sites were excluded if one or more SC lines failed to pass this filter. We considered a site to be polymorphic if one or more SC lines did not contain a copy of the TE. Genotype-specific TIPs (i.e., singletons) were defined as sites where only one SC line contained the TE. Population-specific TIPs were defined as sites where two or three SC lines from the same population contained the TE while the remaining SC lines did not contain the TE.

*TE Mutation Rate Estimation in MA Lines*

We used TEFLoN v0.4 [6] to identify active TEs in the MA lines. For each genotype, we first identified heterozygous sites in all lines (SC, MA, EC) where a site was supported by ≥ 20 reads (presence + absence) with mapping quality ≥ 30 and contained at least one presence and one absence read. For this set of heterozygous sites, we recorded the 10^th^ and 90^th^ percentile of the presence allele frequency (p), denoted as p_10_ and p_90,_ respectively. The median of p in each genotype was less than 0.5, suggesting that presence reads were less likely to map correctly. The mean p_10_ and p_90_ across genotypes were 0.11 and 0.76, respectively. Subsequently, for all sites where all lines (SC, MA and EC) were supported by ≥ 20 presence + absence reads, we assigned a particular line as homozygous for the presence allele if p > 0.95, homozygous for the absence allele if p < 0.05, heterozygous for the presence allele if p_10_ ≤ p ≤ p_90_ and otherwise as undefined. All sites containing one or more undefined lines were discarded from further analyses.

There are two types of TE gain mutations (0-->1 and 1-->2) and two types of TE loss mutations (2-->1 and 1-->0) that can be observed based on whether the ancestor (SC) was homozygous, heterozygous or lacked a TE (an “absence allele”) at a given site relative to the status in the descendant MA line (e.g., if the SC was heterozygous and experienced a gain, it would be classified as a 1-->2 gain event in the MA line; Fig 1). Our ability to detect these different events is not uniform, however, because the likelihood of detecting heterozygous and homozygous “presence” alleles is not simply doubled. Furthermore, because we observe that “presence alleles” in heterozygotes tend to have a frequency below 0.5, it is possible that a line we determined to be homozygous for absence alleles may actually be a heterozygote, but we only observed absence alleles because the presence allele had a higher mapping error rate. To help guard against these types of false positives, we applied an additional filter to the sites tentatively identified to have experienced a TE mutation event. For sites that tentatively experienced a 0-->1 gain or 2-->1 loss, we wanted to guard against the possibility that one or more of the homozygous non-focal lines were actually heterozygous but assigned homozygous due to mapping error. We estimated the frequency of presence and absence alleles for heterozygotes at the site using the frequency in the focal mutant heterozygous line. Given this frequency, we calculated the binomial probability that one or more of the homozygous lines were heterozygous and discarded the site if this probability was larger than 0.005. For sites that tentatively experience a 1-->2 gain or 1-->0 loss, we wanted to guard against the possibility that the focal mutant homozygous line was actually heterozygous but assigned homozygous due to mapping error. To estimate the frequency of presence and absence alleles for heterozygotes at the site, we took the average of frequencies across the heterozygous non-focal lines. Given this averaged frequency, we calculated the binomial probability that the homozygous focal line was heterozygous and discarded the site if this probability was larger than 0.005.

Family-specific mutation rates for each of the four mutation types were calculated using N_m_ / (N_SC_*G), where N_m_ represents that number of sites that experienced a particular mutation event, N_SC_ represents the initial copy number of that TE family in the SC line, and G represents the number of MA generations. Because many repeats cannot be characterized to the level of family, we also ran TEFLoN using the full library containing all known and unknown repeats to obtain the total number of gains and losses and calculate an overall (not family-specific) rate of gain and loss for all repeats found in the initial RepeatModeler run (S23 and S24 Tables), however our downstream analyses focus on the rates calculated for those TEs that could be characterized.

*Estimating false discovery and false omission rates*

The false discovery rate (FDR) represents the proportion of detected mutations that are false positives, while the false omission rate (FOR) represents the proportion of unmutated TE sites that are false negatives. To estimate FDR and FOR, we simulated the MA experiment and processed the data using TEFLoN v0.4 [6] and our filtering pipeline (similar to [6,8]). For each simulation, we generated 11 unique diploid individuals by simulating SNPs onto the largest contig of the FASC assembly (6.8 Mb) using pIRS v1.1.1 [9]. The 11 individuals represented one ancestral line and 10 descendent (mutation accumulation + extant control) lines. We then inserted 50 of one type of TE mutation event: 0-->1 gain, 1-->0 loss, 1-->2 gain or 2-->1 loss. For each mutation, we randomly picked an existing TE on the contig with a minimum length of 400 bp and randomly inserted it back into the contig, making sure it did not overlap with other TEs. We included a target site duplication (TSD) flanking each insertion with a mean length of 5 bp drawn from a Poisson distribution. To simulate a 0-->1 gain, we inserted a heterozygous TE (i.e., on one homolog of the contig) into one of the descendent lines. To simulate a 2-->1 loss, we inserted a homozygous TE (i.e. insertion of both homologs) on the ancestral and all non-focal descendant lines and then inserted a heterozygous TE on the focal MA line. To simulate a 1-->2 gain, we inserted a heterozygous TE on the ancestral and all non-focal descendant lines and then inserted a homozygous TE on the focal MA line. Lastly, to simulate a 1-->0 loss, we inserted a heterozygous TE on the ancestral and all non-focal descendant lines and did not change the focal descendent line. Finally, we independently simulated pair-end reads for each of the 11 individuals with an average coverage of 50x using pIRS [9] and filtered for mutations in the same way as described above. We utilized 50x as the sequencing depth because the median depth of coverage across all our SC, MA, and EC lines was approximately 50, on average (S26 Table).

For each mutation type, we repeated the simulation four times. We also examined whether the minimum length of TEs affected FPR and FNR by repeating the whole process and setting the minimum length of TEs to 800 bp. In total, we simulated 400 of each mutation type. FDR for focal mutation type was estimated as FP / (FP + TP), where FP is the number of discovered TEs falsely inferred to be the focal mutation type, and TP is the number of simulated mutations that were detected. The FOR for the focal mutation type was estimated as FN / (FN + TN), where FN is the number of simulated mutations that were not identified and TN is the number of existing TEs not identified as a mutation. Although we do not incorporate the FDR and FOR into the reported rates our main analysis, we provide rates adjusted for the FDR in S13 Table.

*Statistical Analyses*

To test if TE abundance differed intraspecifically, we performed a two-way ANOVA on the log of TE percent abundance with genotype and TE family as fixed factors. We performed this analysis on TE abundance estimated from the RepeatMasker [7] and the read mapping approach. Similarly, we performed a two-way ANOVA on the log of TE percent abundance with species and TE family as fixed factors to examine interspecific variation between *D. magna* and *D. pulex*. For this analysis, we restricted the TE families to those that were present in both species and only the estimates from RepeatMasker [7] were utilized. Lastly, we examined if the total percentage abundance of TEs in *D. magna* was significantly different from the abundance in *D. pulex* including all TE families, not just the shared TE families. This was performed by applying a one-sample t-test on the TE abundance of *D. magna* to test if the mean across the 9 genotypes differed from the abundance of *D. pulex* (which we only have one estimate for TE abundance).

We also examine intraspecific and interspecific differences in pairwise divergence of TE families. For each TE family, we performed a one-way ANOVA to test whether pairwise divergence varied among the nine genotypes of *D. magna* and a two-sample t-test to test whether pairwise divergence differed between *D. magna* and *D. pulex*. To examine if mutational activity in the MA experiment is reflected in the pairwise divergence observed in natural isolates (SC lines), we performed a two-sample t-test on the pairwise divergence of elements in TE families detected to be mutationally active in MA lines against TE families that were not mutationally active. Bonferonni correction was used to adjust P-values for multiple testing.

We utilized principal components analysis (PCA) to visualize variation at TIPs across SC lines. PC axes were used as input for K-means clustering. We varied the number of clusters (k) from 1 to 9 and the optimal number of clusters was determined by the value of k that maximized the average silhouette. This was performed separately for analyses using each of the 9 reference assemblies. Presence and absence of TE for each SC line at polymorphic TE sites are shown in S29 Table (results are for analyses using the FASC assembly as reference).

To test if gain and loss rates differed between MA and EC lines, we fitted a binomial mixed-effects model on mutation rates with treatment (MA or EC) as fixed effects, genotype as a random effect and TE family as a random effect. To test for differences in TE mutation rates across populations, we used a binomial mixed-effects model on the rates of gains (or losses) with population as the fixed effect, genotype nested within population as a random effect and TE family as a random effect. We then applied post-hoc Tukey HDS tests to determine which pairs of population differed significantly in gain and loss rates. We used Pearson correlations to test for statistical association among rates for different types of mutations. Base substitution mutation rates and gene conversion rates were obtained from a previous study on the same *D. magna* MA lines [10]. All code for data processing and analysis is available at <https://github.com/EddieKHHo/DaphiaMagna_MA_TE> and sequence data have been deposited at NCBI (PRJNA658680).

**References**

1. Bushnell B, Rood J, Singer E. BBMerge – Accurate paired shotgun read merging via overlap. PLOS ONE. 2017;12: e0185056. doi:[10.1371/journal.pone.0185056](https://doi.org/10.1371/journal.pone.0185056)
2. Bankevich A, Nurk S, Antipov D, Gurevich AA, Dvorkin M, Kulikov AS, et al. SPAdes: A new genome assembly algorithm and its applications to single-cell sequencing. J Comput Biol. 2012;19: 455–477. doi:[10.1089/cmb.2012.0021](https://doi.org/10.1089/cmb.2012.0021)
3. Pryszcz LP, Gabaldón T. Redundans: an assembly pipeline for highly heterozygous genomes. Nucleic Acids Res. 2016;44: e113–e113. doi:[10.1093/nar/gkw294](https://doi.org/10.1093/nar/gkw294)
4. Li H, Durbin R. Fast and accurate short read alignment with Burrows-Wheeler transform. Bioinformatics. 2009;25: 1754–1760. doi:[10.1093/bioinformatics/btp324](https://doi.org/10.1093/bioinformatics/btp324)
5. Fu L, Niu B, Zhu Z, Wu S, Li W. CD-HIT: accelerated for clustering the next-generation sequencing data. Bioinformatics. 2012;28: 3150–3152. doi:[10.1093/bioinformatics/bts565](https://doi.org/10.1093/bioinformatics/bts565)
6. Adrion JR, Begun DJ, Hahn MW. Patterns of transposable element variation and clinality in *Drosophila*. Mol Ecol. 2019;28: 1523–1536. doi:[10.1111/mec.14961](https://doi.org/10.1111/mec.14961)
7. Smit AFA, Hubley R, Green P. RepeatMasker Open-4.0. 2013. Available: <http://www.repeatmasker.org>
8. Adrion JR, Song MJ, Schrider DR, Hahn MW, Schaack S. Genome-wide estimates of transposable element insertion and deletion rates in *Drosophila melanogaster*. Genome Biol Evol. 2017;9: 1329–1340. doi:[10.1093/gbe/evx050](https://doi.org/10.1093/gbe/evx050)
9. Hu X, Yuan J, Shi Y, Lu J, Liu B, Li Z, et al. pIRS: Profile-based Illumina pair-end reads simulator. Bioinformatics. 2012;28: 1533–1535. doi:[10.1093/bioinformatics/bts187](https://doi.org/10.1093/bioinformatics/bts187)
10. Ho EKH, Macrae F, Latta LC, McIlroy P, Ebert D, Fields PD, et al. High and highly variable spontaneous mutation rates in *Daphnia*. Molecular Biology and Evolution. 2020;37: 3258–3266. doi:[10.1093/molbev/msaa142](https://doi.org/10.1093/molbev/msaa142)
