# Supplementary material for: Engines of change: Transposable element mutation rates are high and variable within Daphnia magna
Source: PLoS Genet. 2021 Nov 1;17(11):e1009827. doi: 10.1371/journal.pgen.1009827 (PMC8594854; doi:10.1371/journal.pgen.1009827)
Supplement: S2 Text — (DOCX) [file pgen.1009827.s002.docx]

**S2 Text: Supplementary Results**

*Repeat masking versus read mapping to estimate TE abundance*

The disparity between methods for estimating TE abundance (repeat masking versus read mapping) is likely because more repetitive genomes are harder to assemble, and the estimation of repeat content using repeat masking depends on the quality of the assembly. In support of this notion, we observe a negative correlation between assembly size and proportion of the assembly containing TEs using a read mapping approach (ρ = -0.69, t_7_ = -2.49, P = 0.042), as would be expected if high repeat content impedes confident assembly. The abundance estimates based on the two methods differ considerably if you aggregate by TE type (ρ = -0.19, t_7_ = -0.49, *p* = 0.63), however when compared by individual TE family the methods correlate strongly (bp averaged across genotypes; ρ = 0.99, t_33_ = 34.1, *p* < 0.0001; S5 Table).

*Analysis of TE insertion site polymorphism using different reference assemblies*

When searching for TE insertion site polymorphisms (TIPs), we had to choose a common reference assembly for TEFLoN to map reads against. Since we had nine reference assemblies, we ran this analysis nine times using each of the different assemblies as reference. Although the total number of TIPs found varied when using different assemblies (S7 Table), the general patterns are robust across the nine analyses. Furthermore, the genetic relatedness among the 9 genotypes was calculated by calculating mean pairwise genetic distances using single nucleotide polymorphism data from [1] to ensure differences among genotypes in terms of TEs were not just a by-product of genetic distance (S28 Table).

Firstly, we found no significant difference for the distribution of TIPs in each TE family (𝝌2 = 238.51, df = 240, P = 0.51; S7 Table). The TE families Gypsy, Pao, hAT-Ac, Copia, I, and DIRs possessed the highest number of TIPs across all nine analyses. Secondly, principal components analysis was also able to cluster genotypes by their population of origin regardless of the reference assembly used (S1 Fig). Lastly, neither the distribution for the frequency of TEs at TIPs (𝝌2 = 67.93, df = 72, P = 0.61; S2 Fig) nor the proportion of singletons belonging to each population (𝝌2 = 8.8, df = 16, P = 0.92; S3 Fig) were significantly different when using different reference assemblies. Across the nine analyses, 42-53% of TIPs were population-specific and 13-16% were singletons (S2 Fig). In addition, Israel genotypes tend to possess the highest number of population-specific TEs, while Germany genotypes tend to possess the least. The relative lack of singletons TIPs in German genotypes may help to explain why German genotypes tend to cluster less closely than Finnish and Israeli genotypes on our PCA plots (S1 Fig).

*Using Pairwise Divergence Estimates Among TE Copies to Make Inferences About Activity*

Frequency histograms of pairwise divergences or means (MPD) are often used to infer when TE families were active in the genome (lower values suggest more recent invasion/activity because there has been less time to accrue point mutations among copies). There was a wide range of MPDs observed across TE families (14.6% to 30.8%; S11 Table; S4 - S15 Figs). While 21 out of 26 TE families differ in pairwise divergence between *D. magna* and *D. pulex*, they are not consistently higher in one species compared to the other (8 and 13 families have lower and higher MPD in *D. magna*, respectively; S12 Table; S4 – S15 Figs).

*Rate estimates including unclassified (“unknown”) repeats*

Our main analysis excluded a large number of interspersed repeats that were designated as “unknown” by RepeatModeler v1.0.11 [2]. To examine if these unknown repeats experienced mutational events, we ran our analysis again using a repeat library containing the known TEs from the main analysis and the unknown repeats. The analysis including the unknown repeats discovered 225 indels, which was greater than the 95 events in the main analysis (S16 Table). Of the 225 indels, 72 were associated with TEs and 153 with unknown repeats.

The effect of including unknown repeats in our analysis varied greatly among genotypes. While we observed a modest increase in mutations for GC, IA and IB, there was a larger increase of 29 and 57 events for FC and GB, respectively (S17 Table). Furthermore, we found 0 mutations for FB in our main analysis but our analysis with unknown repeats discovered 5 mutations. Interestingly, the distribution of gain and losses were relatively similar between TE and unknown repeats for FC and GB. In FC, we observed a bias for 0-->1 gains for both TE and unknown repeats while for GB the bias was for 1-->0 losses. Although these results suggest we may be underestimating the total count of TE mutations, the total rate of mutations was similar to our main analysis that excluded unknown repeats (S17 Table). In fact, the total rate was slightly lower for FC, GC and IB in the analysis that included the unknown repeats. This is reassuring because it suggests that some mutational properties of TEs can be approximated even if many TEs have not been fully characterized.

*Simulations to estimate false positive and false negative rates*

We used simulations to estimate the false positive and false negative rate (FPR and FNR) for the four cases of events (Fig 1). Two categories of events (0-->1 gains and 2-->1 losses) have low FNRs (0.06 and 0.09, respectively), while 1-->2 gains and 1-->0 losses had higher FNRs (0.13 and 0.28, respectively; S18 Table). We determined that the higher FNRs for 1-->2 gains and 1-->0 losses were mainly due to the heterozygous TE sites not being detected by TEFLoN, possibly due to low coverage and/or mapping errors for repetitive regions. False positive rates (FPR) were relatively low (< 0.013) for all four types of mutations (S16 Table), and neither FNRs and FPRs varied depending on TE length (S18 Table).

In addition to our simulations, we tested for a correlation between median depth of coverage for each genome sequences for each lineage (S26 Table) and the number of TE insertions detected, and found no significant correlation (S28 Table).

**References**

1. Ho EKH, Macrae F, Latta LC, McIlroy P, Ebert D, Fields PD, et al. High and highly variable spontaneous mutation rates in *Daphnia*. Singh N, editor. Molecular Biology and Evolution. 2020;37: 3258–3266. doi:[10.1093/molbev/msaa142](https://doi.org/10.1093/molbev/msaa142)
2. Smit AFA, Hubley R. RepeatModeler Open-1.0. 2008. Available: <http://www.repeatmasker.org>
