# Supplementary figures and images for: Engines of change: Transposable element mutation rates are high and variable within Daphnia magna
Source: PLoS Genet. 2021 Nov 1;17(11):e1009827. doi: 10.1371/journal.pgen.1009827 (PMC8594854; doi:10.1371/journal.pgen.1009827)

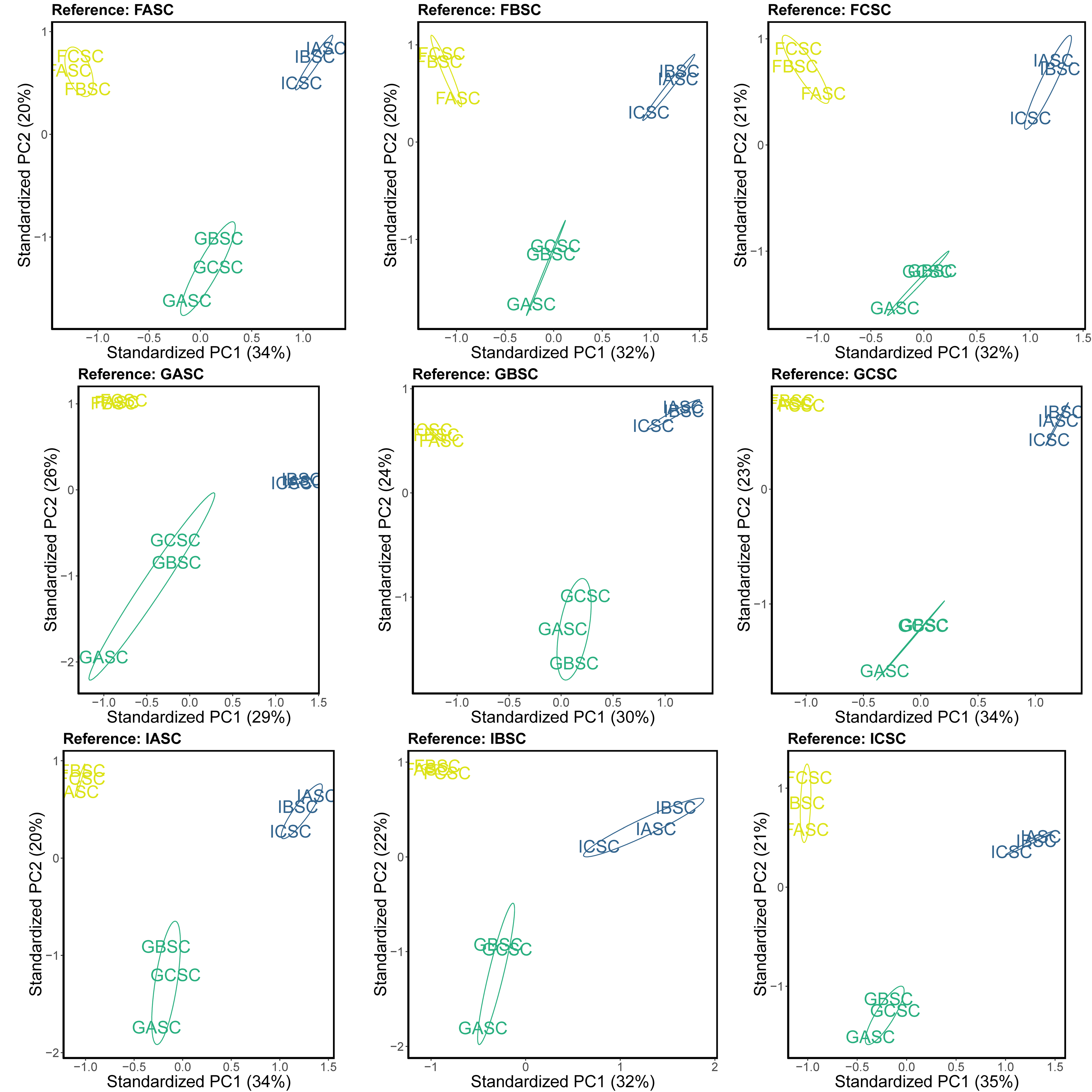

Supplement: S1 Fig — Variance explained by principal components 1 and 2 are displayed on the axes. The reference assembly used is indicated on the top of each plot. Genotypes from Finland, Germany and Israel are colored in gold, green, blue, respectively. (TIF) [file pgen.1009827.s033.tif]

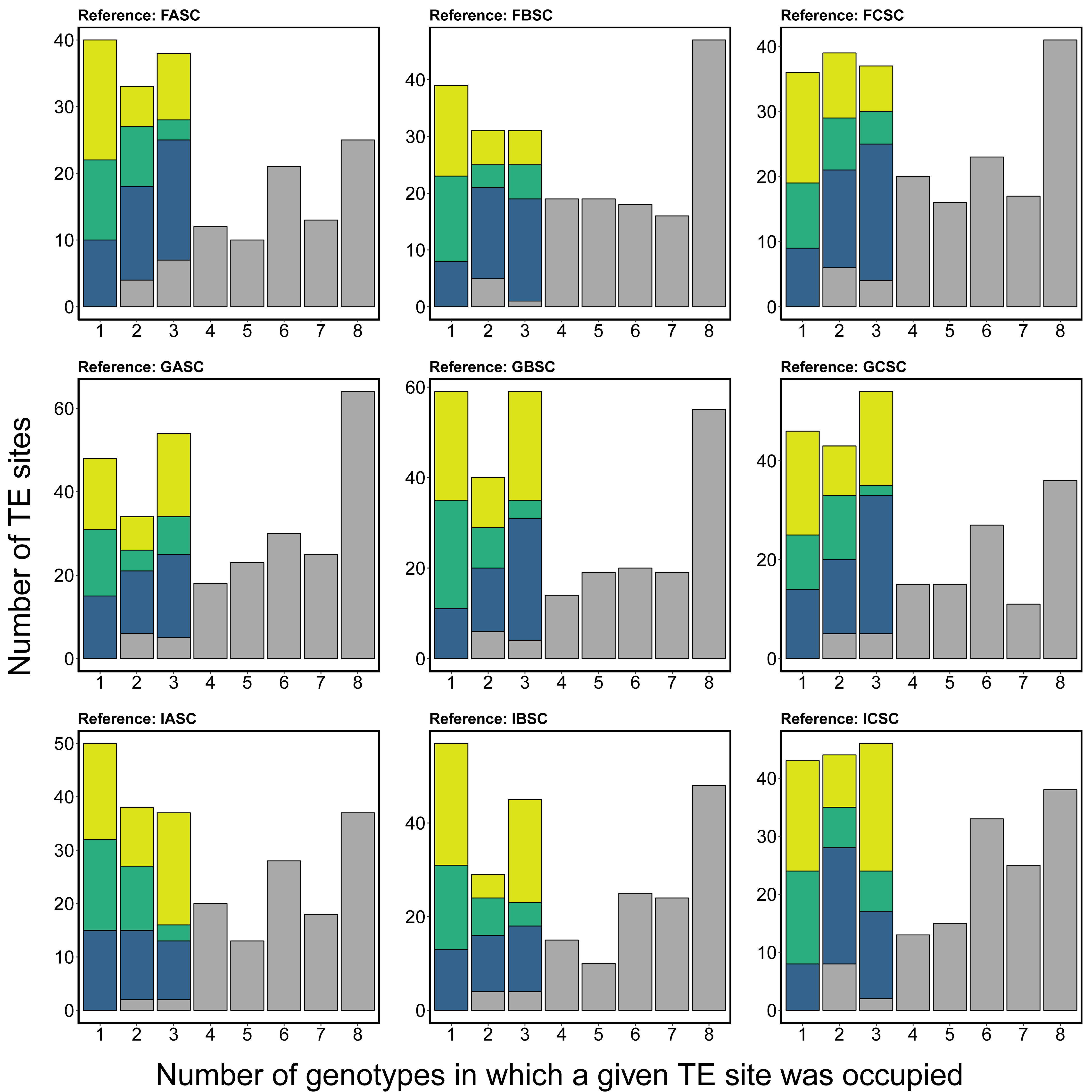

Supplement: S2 Fig — The left bar represents the number of singletons (sites occupied in only one genotype) for each population (gold, green and blue for Finland, Germany and Israel, respectively). Colored portions of bars in x = 2 and x = 3 represent sites occupied in 2 and 3 genotypes, respectively, when from the same population. Grey portions of each bar represent the number of sites that were occupied in ≥2 genotypes that were not population-specific. The reference assembly used is indicated on the top of each plot. (TIF) [file pgen.1009827.s034.tif]

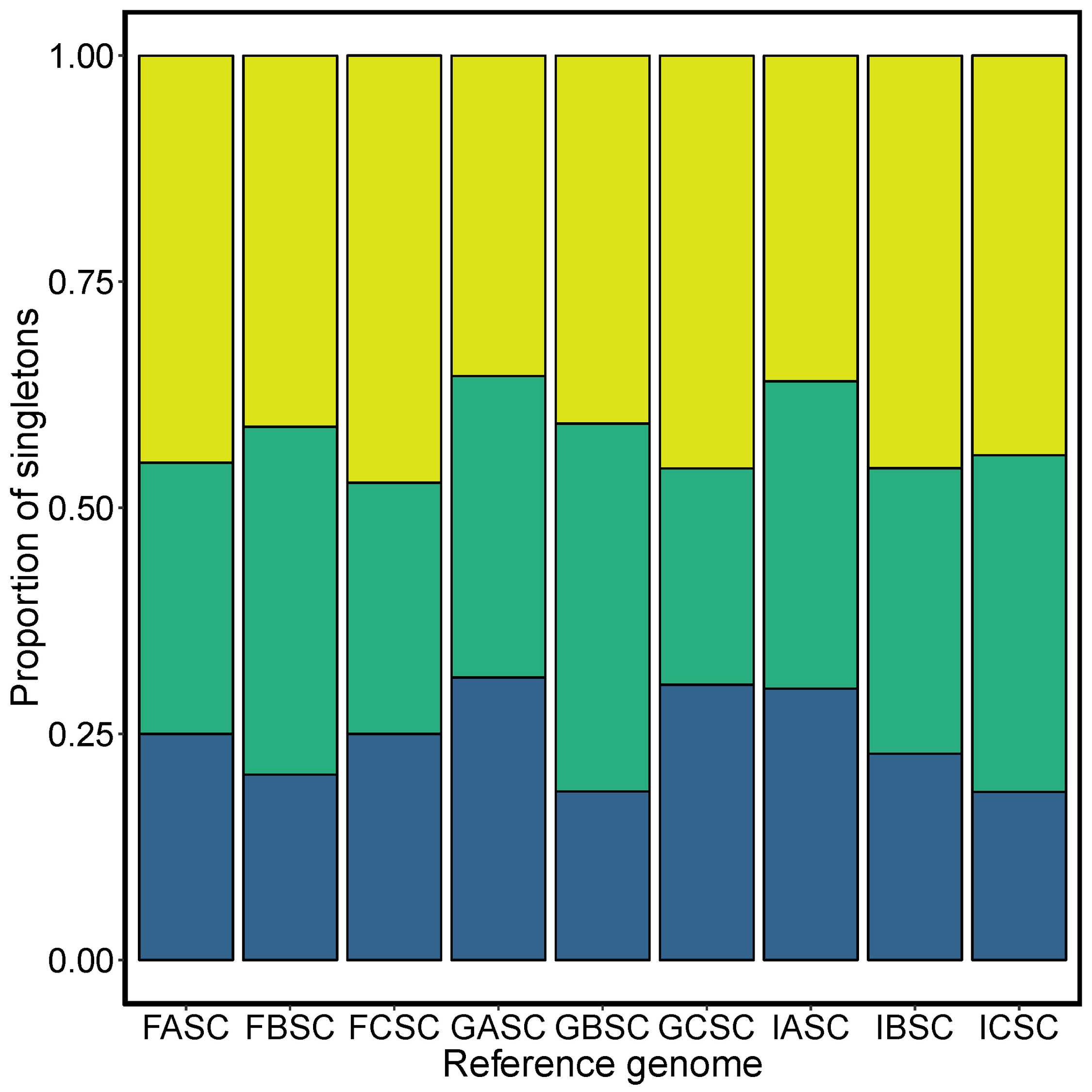

Supplement: S3 Fig — Gold, green and blue represents singletons specific to genotypes in Finland, Germany and Israel, respectively. The proportion of singletons belonging to each population was not significantly different when using different reference genomes (χ2 = 8.8, df = 16, P = 0.92). (TIF) [file pgen.1009827.s035.tif]

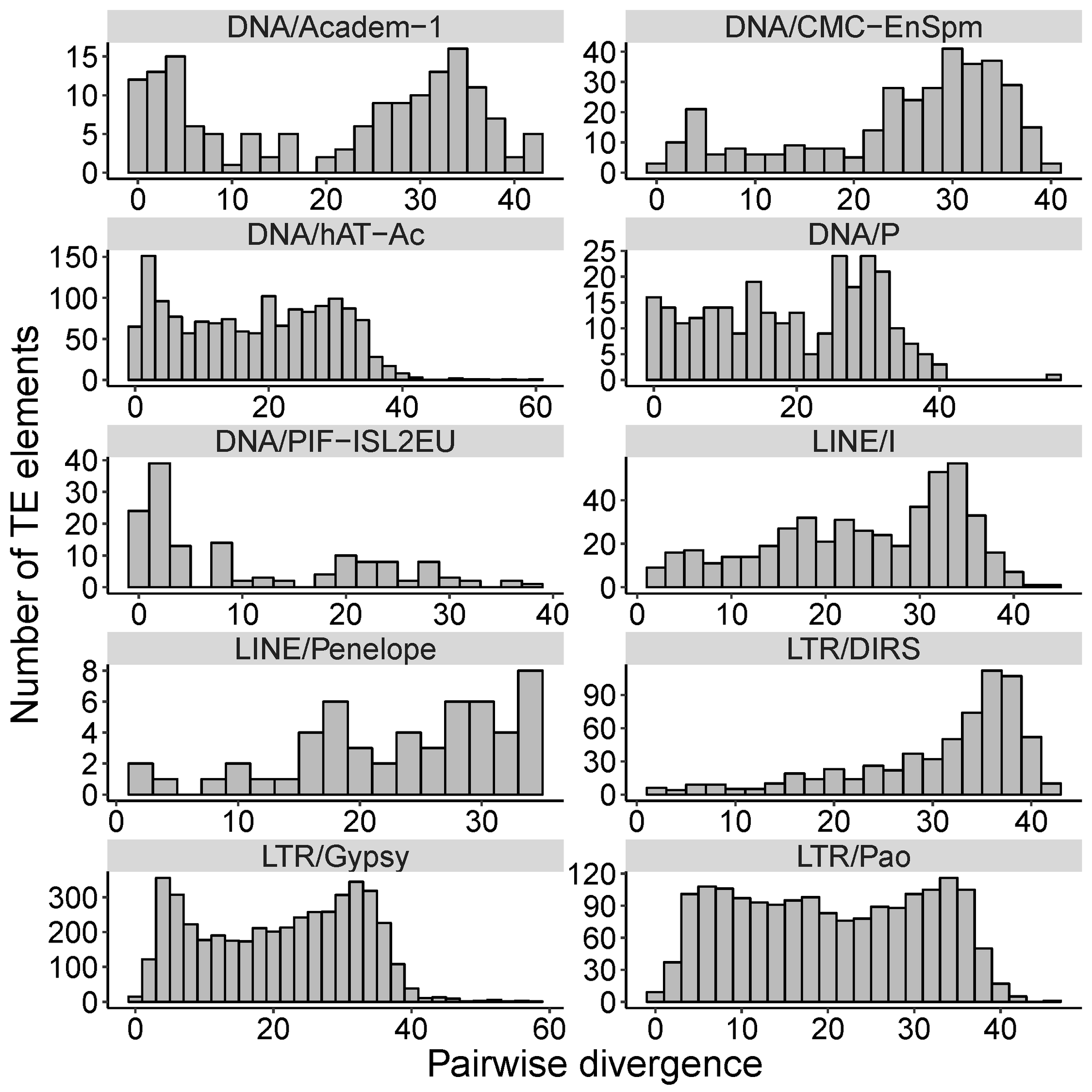

Supplement: S4 Fig — (TIF) [file pgen.1009827.s036.tif]

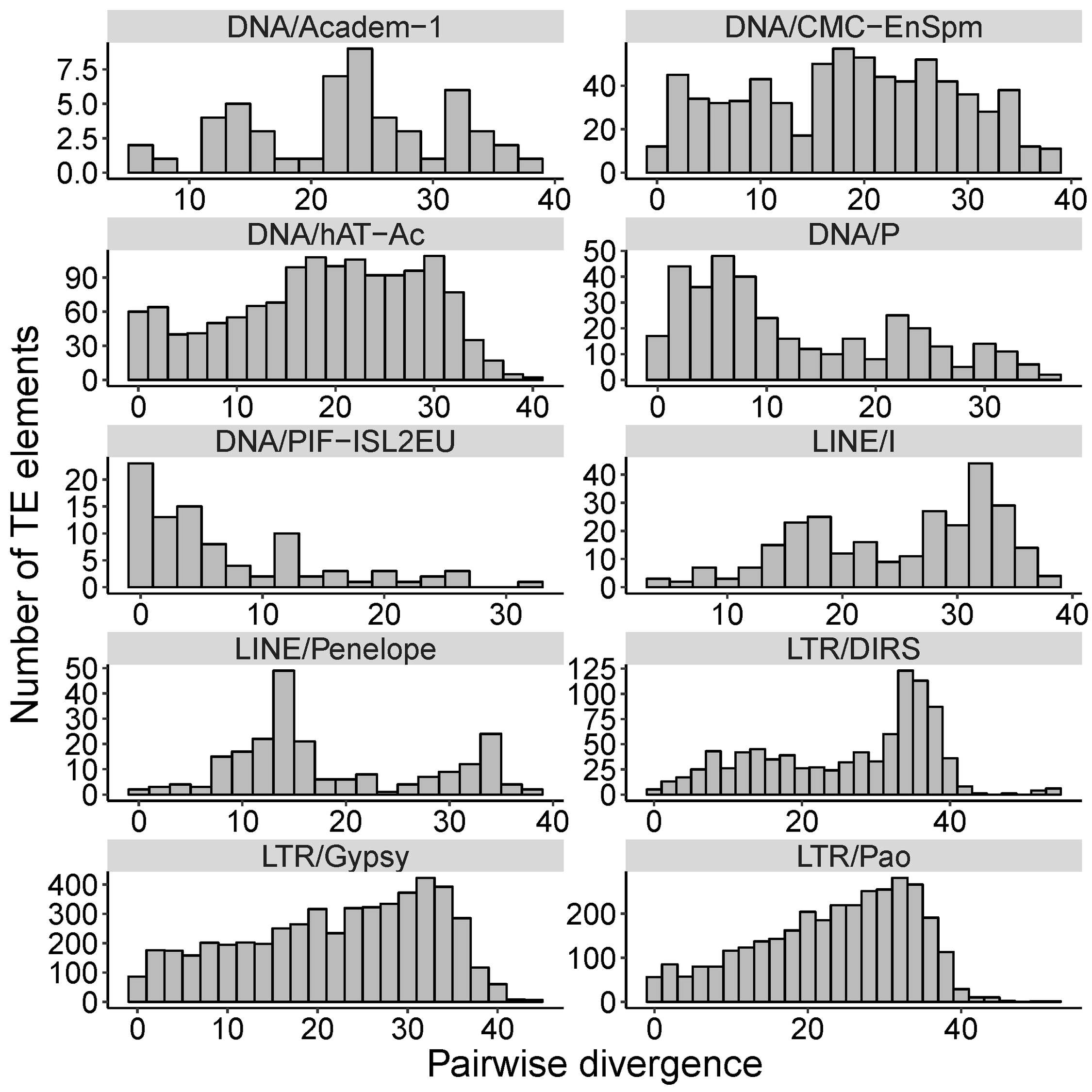

Supplement: S5 Fig — (TIF) [file pgen.1009827.s037.tif]

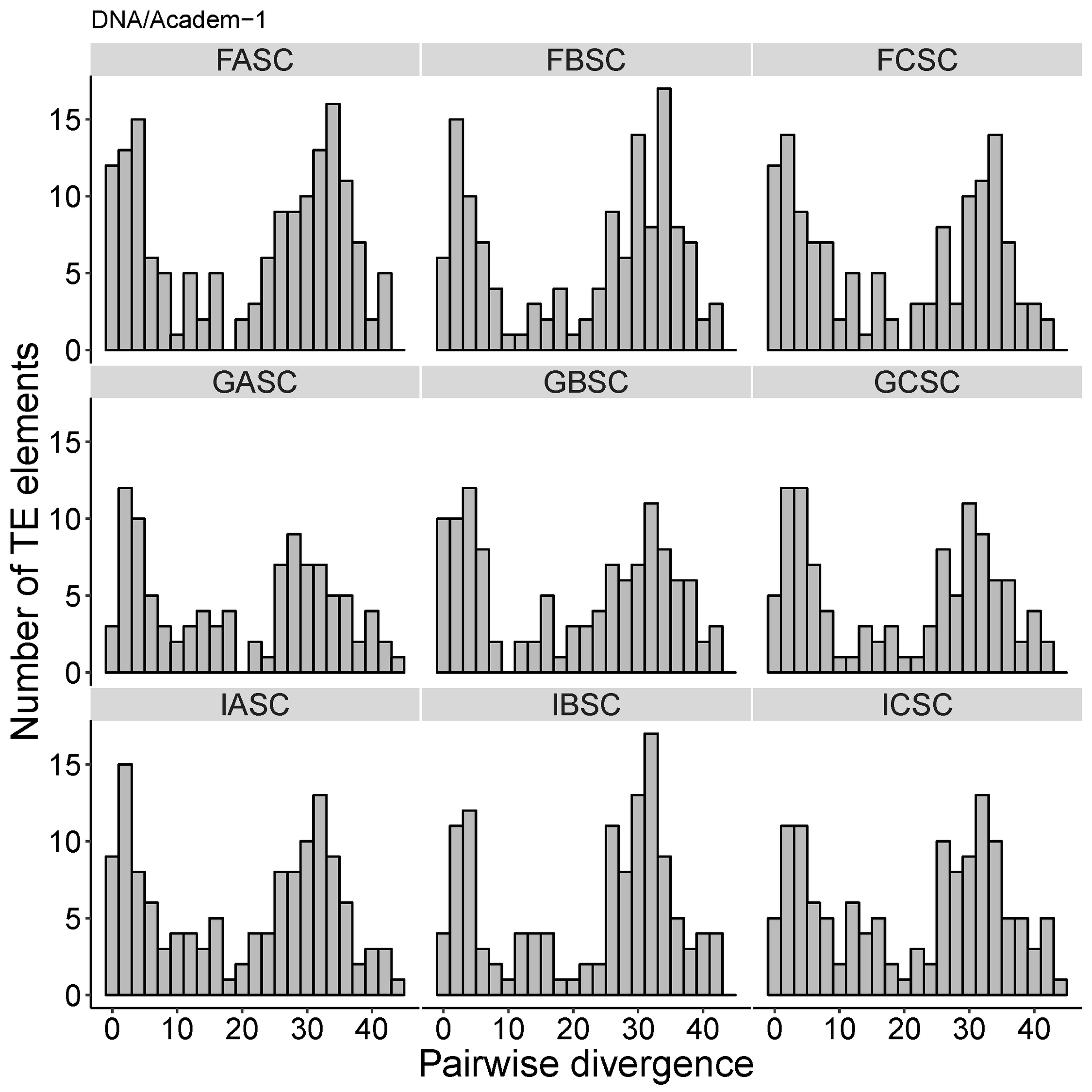

Supplement: S6 Fig — (TIF) [file pgen.1009827.s038.tif]

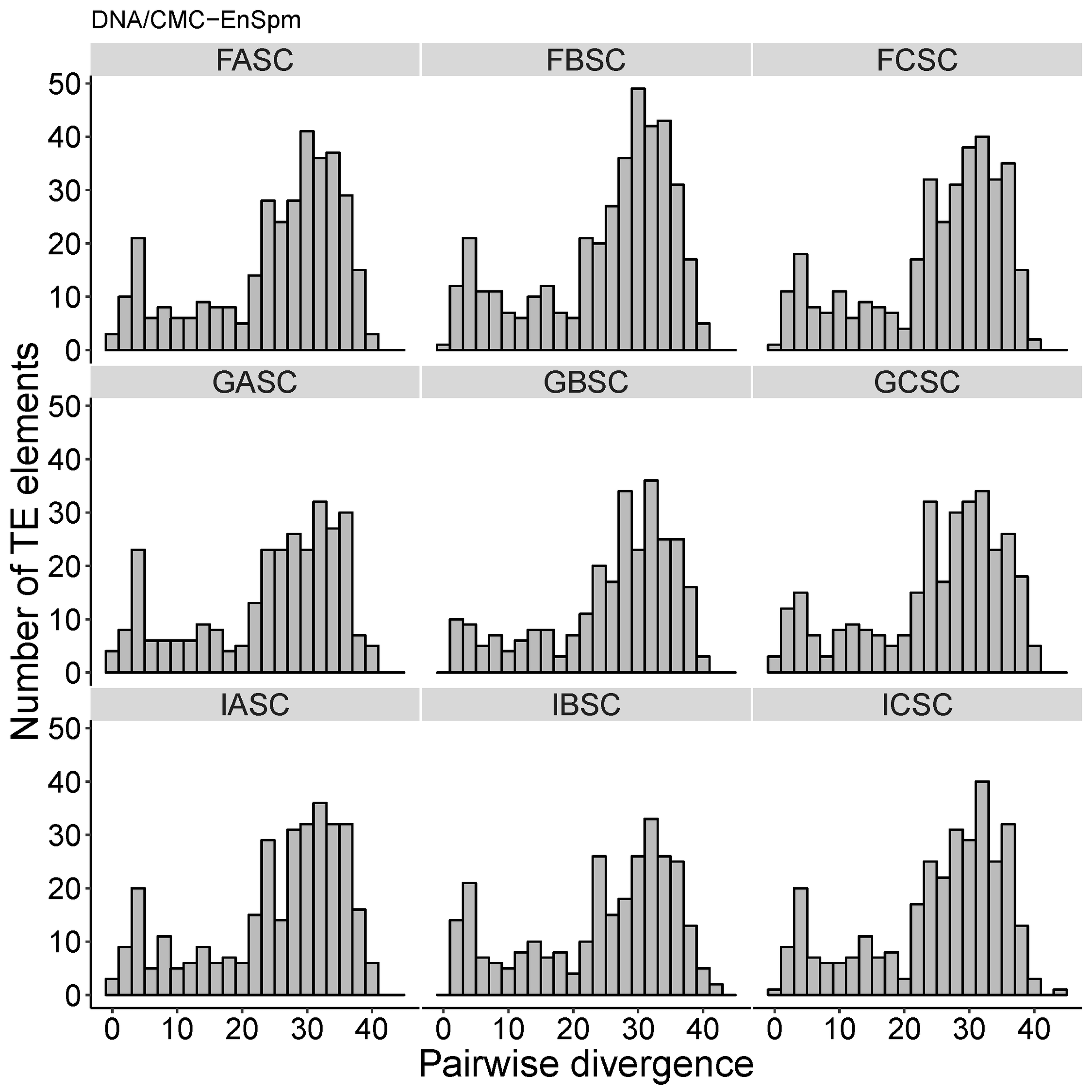

Supplement: S7 Fig — (TIF) [file pgen.1009827.s039.tif]

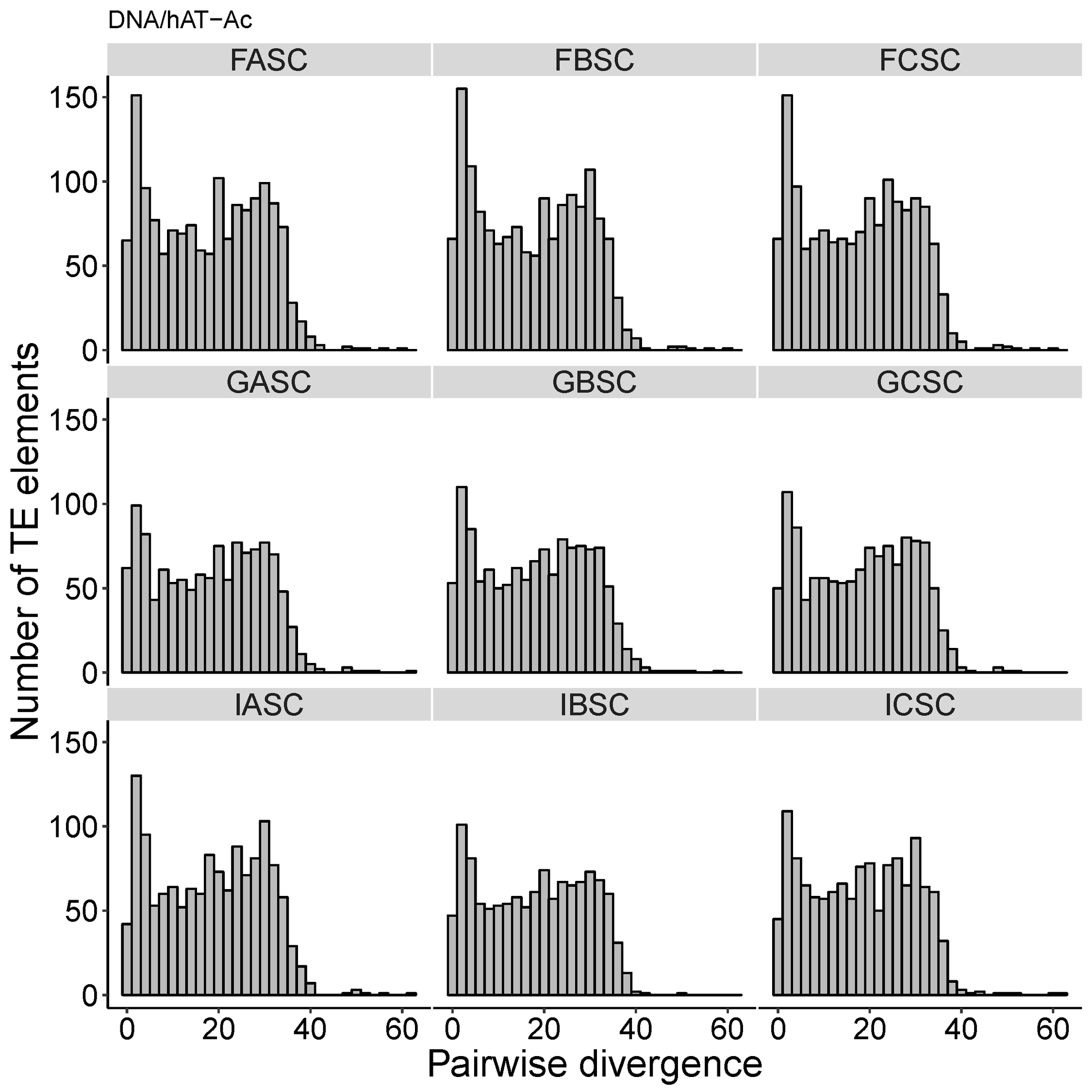

Supplement: S8 Fig — (TIF) [file pgen.1009827.s040.tif]

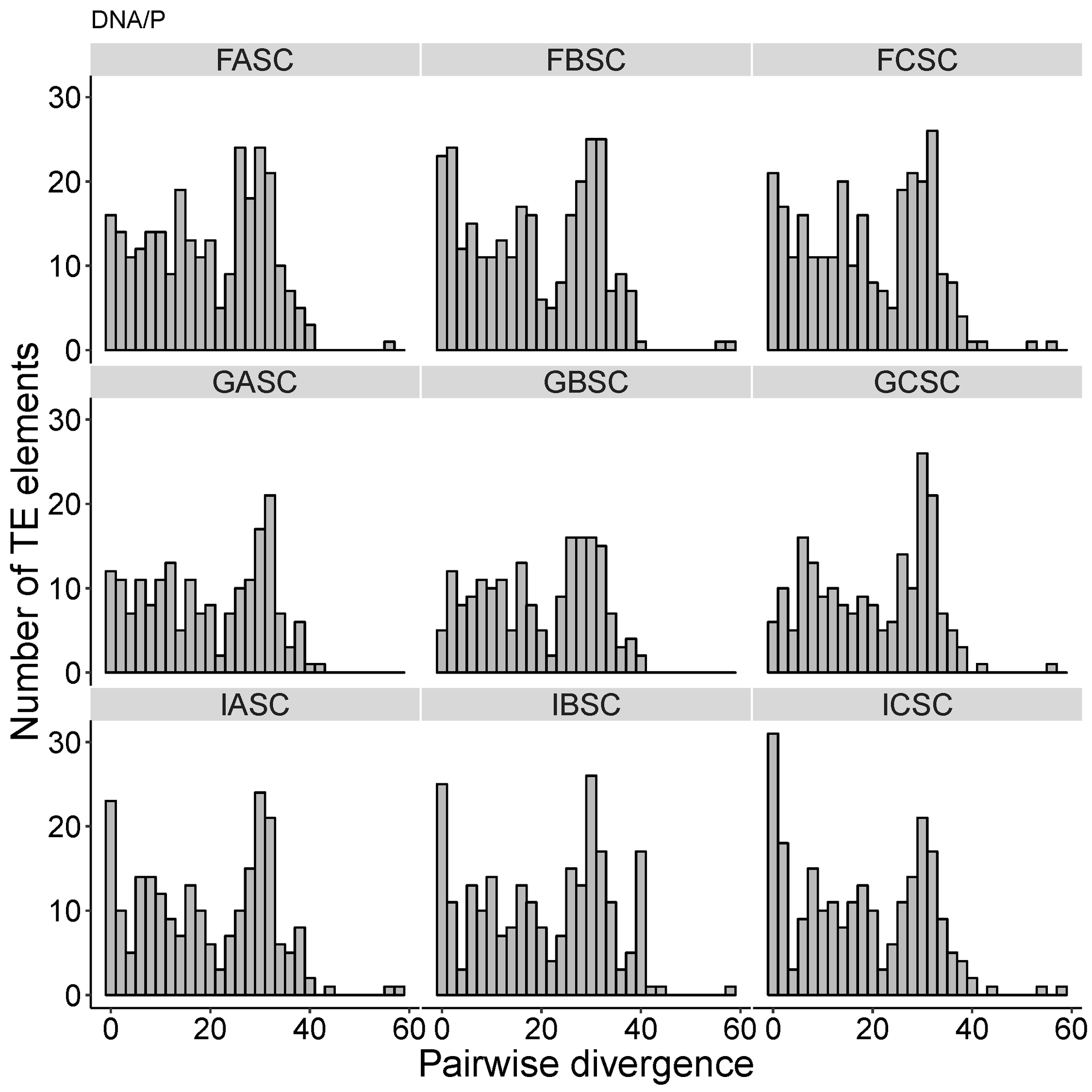

Supplement: S9 Fig — (TIF) [file pgen.1009827.s041.tif]

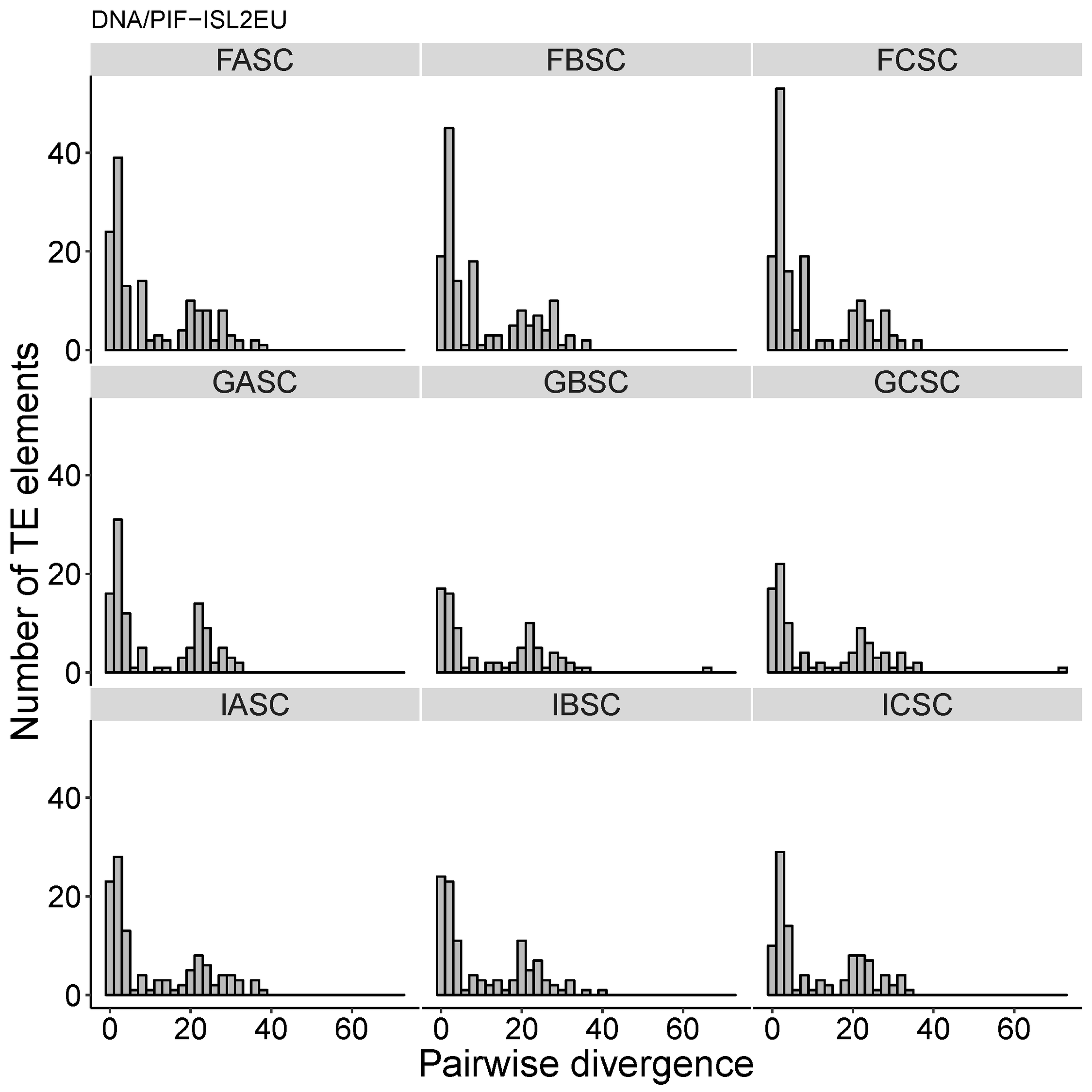

Supplement: S10 Fig — (TIF) [file pgen.1009827.s042.tif]

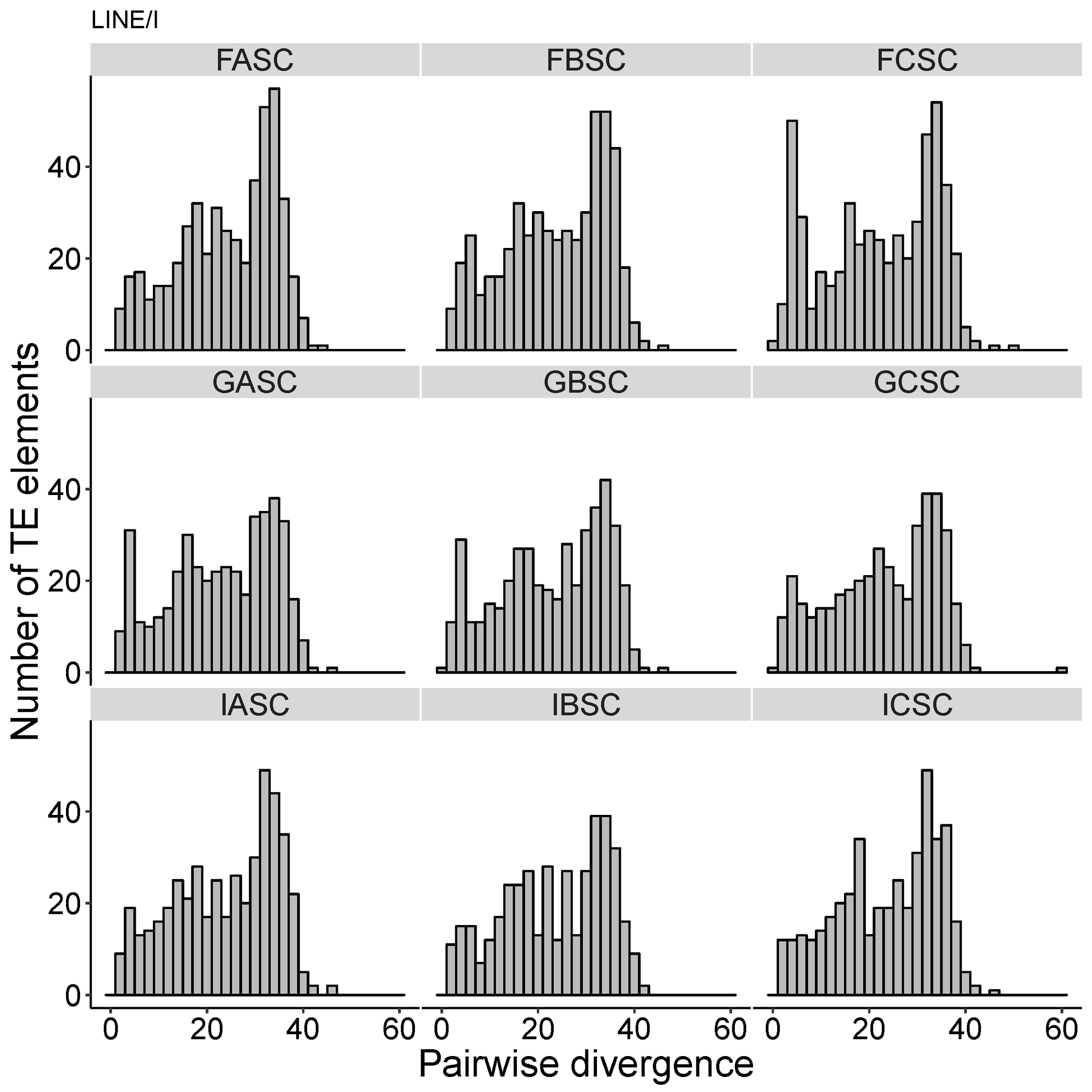

Supplement: S11 Fig — (TIF) [file pgen.1009827.s043.tif]

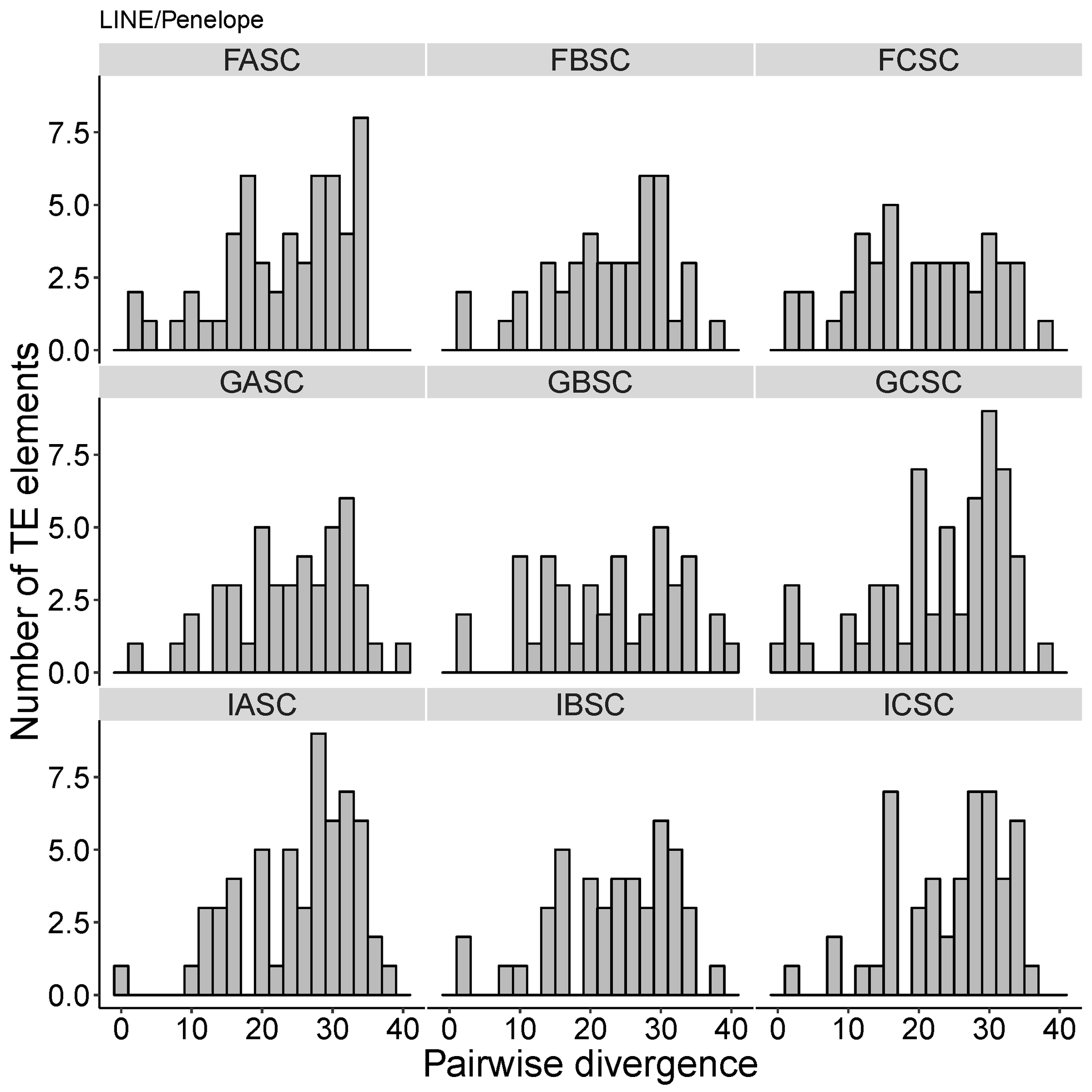

Supplement: S12 Fig — (TIF) [file pgen.1009827.s044.tif]

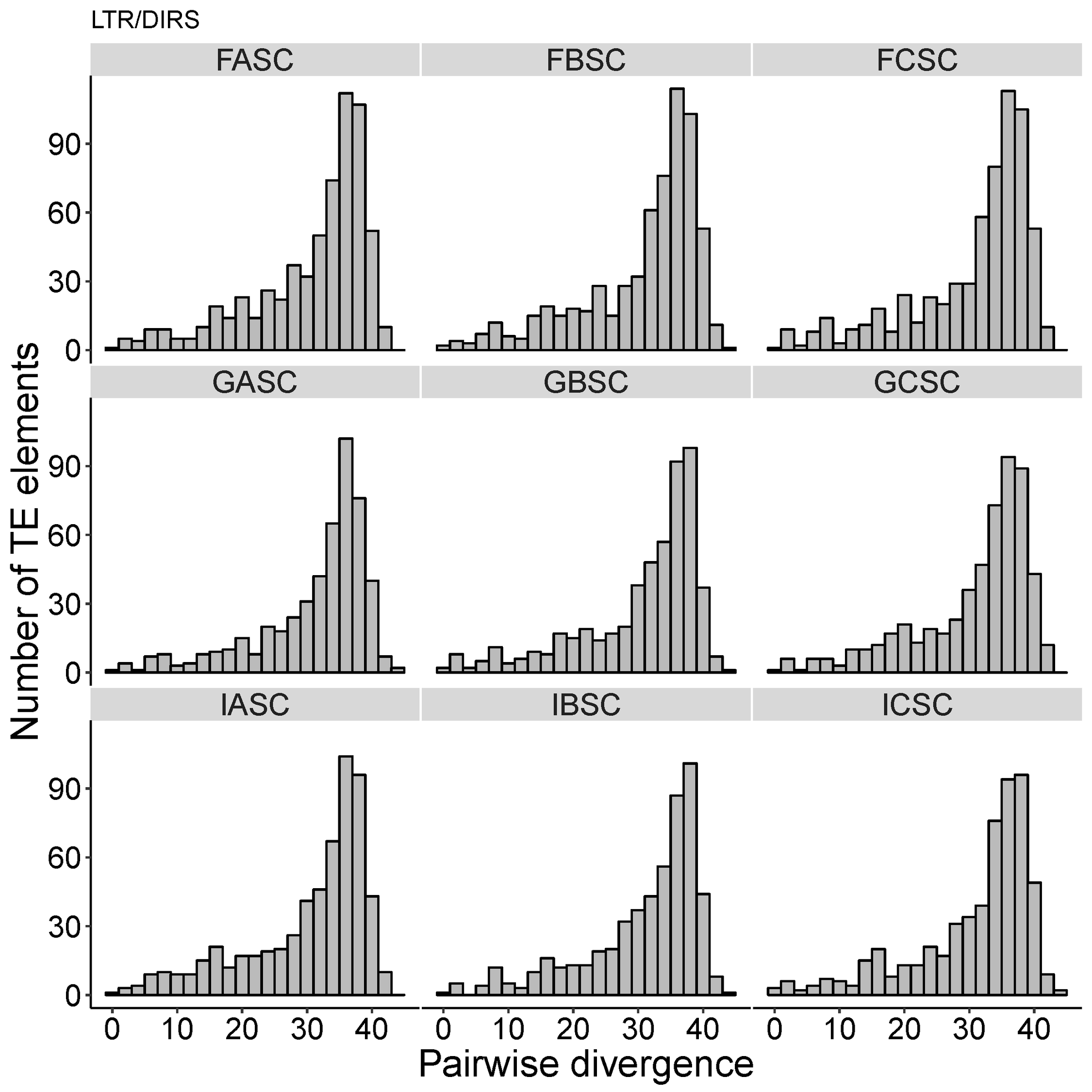

Supplement: S13 Fig — (TIF) [file pgen.1009827.s045.tif]

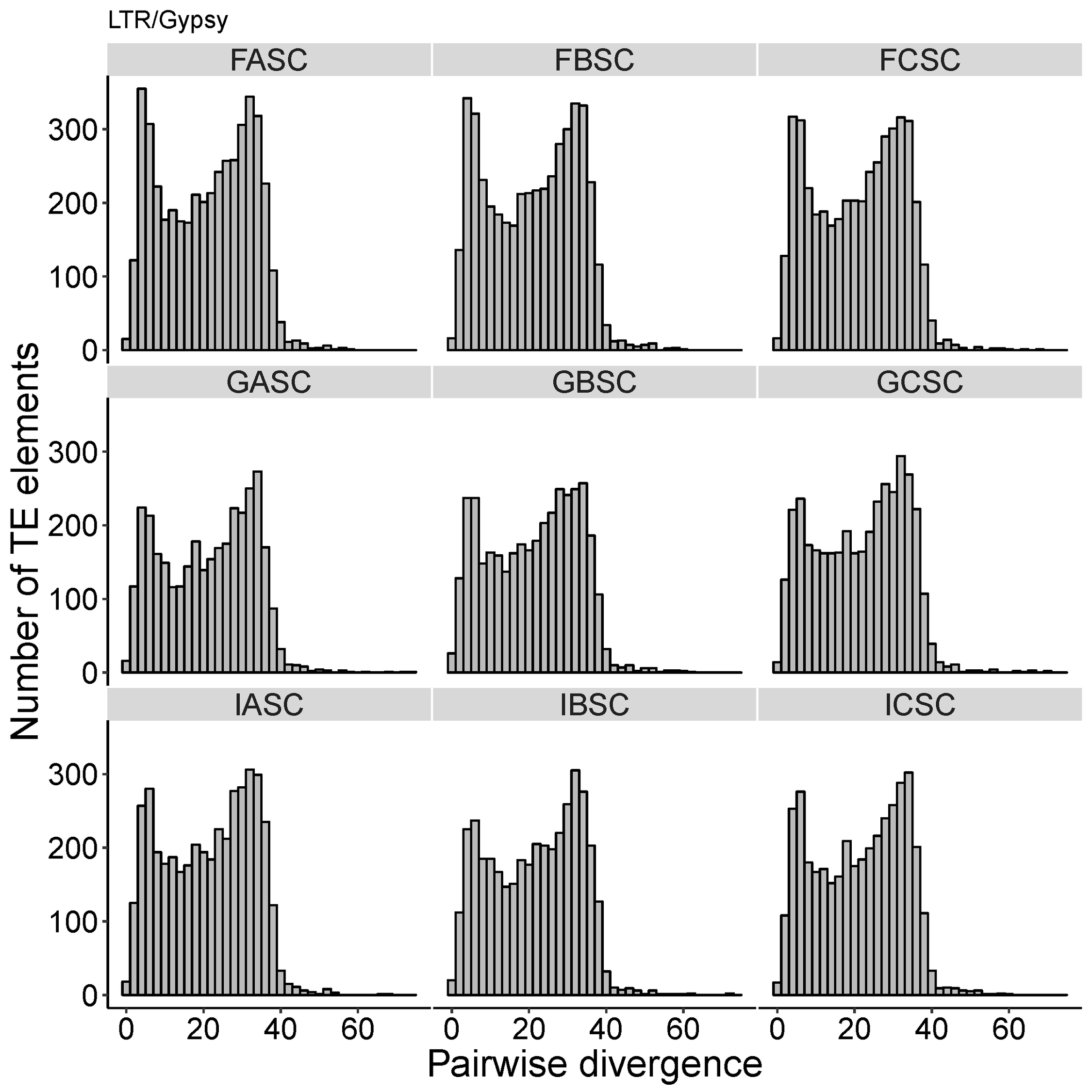

Supplement: S14 Fig — (TIF) [file pgen.1009827.s046.tif]

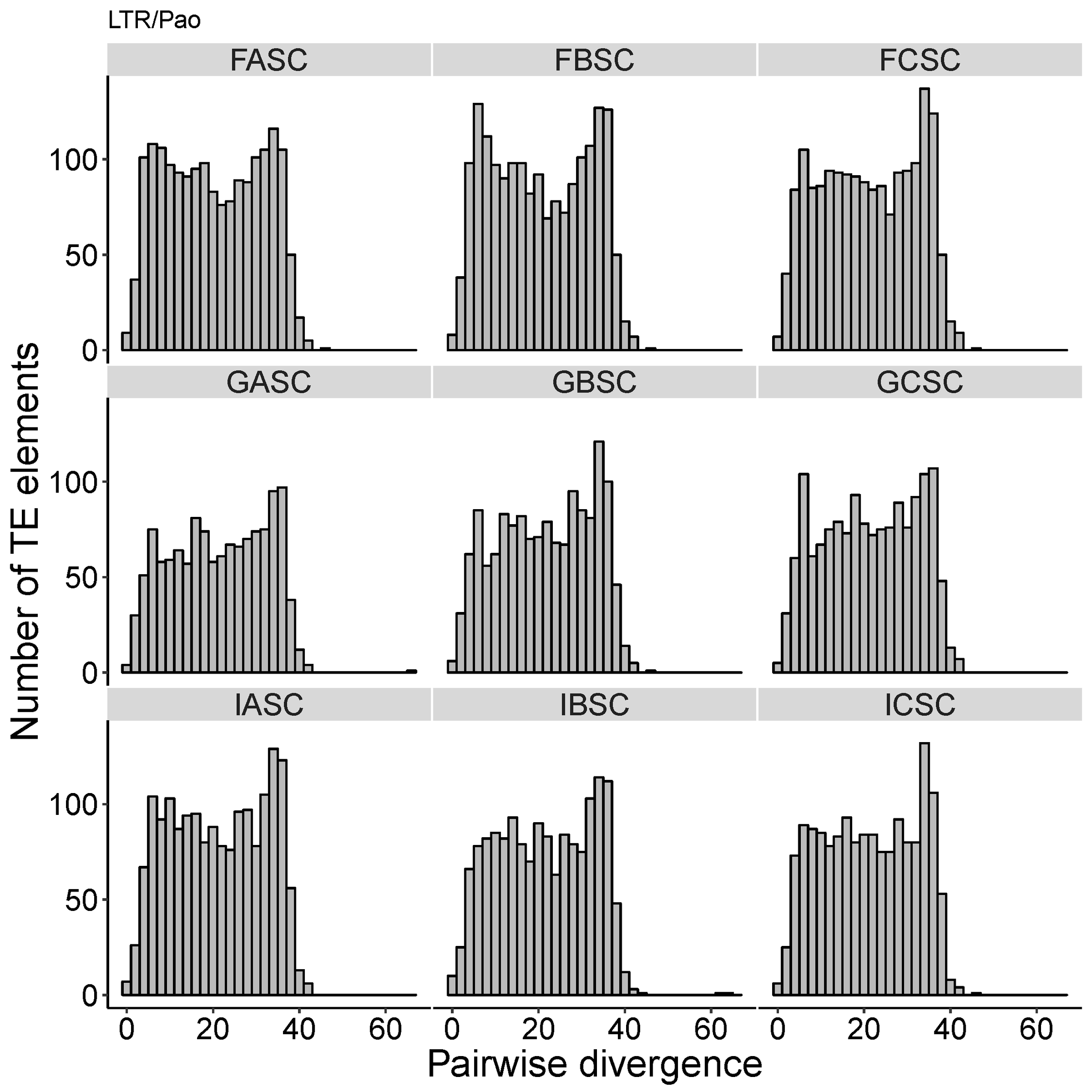

Supplement: S15 Fig — (TIF) [file pgen.1009827.s047.tif]

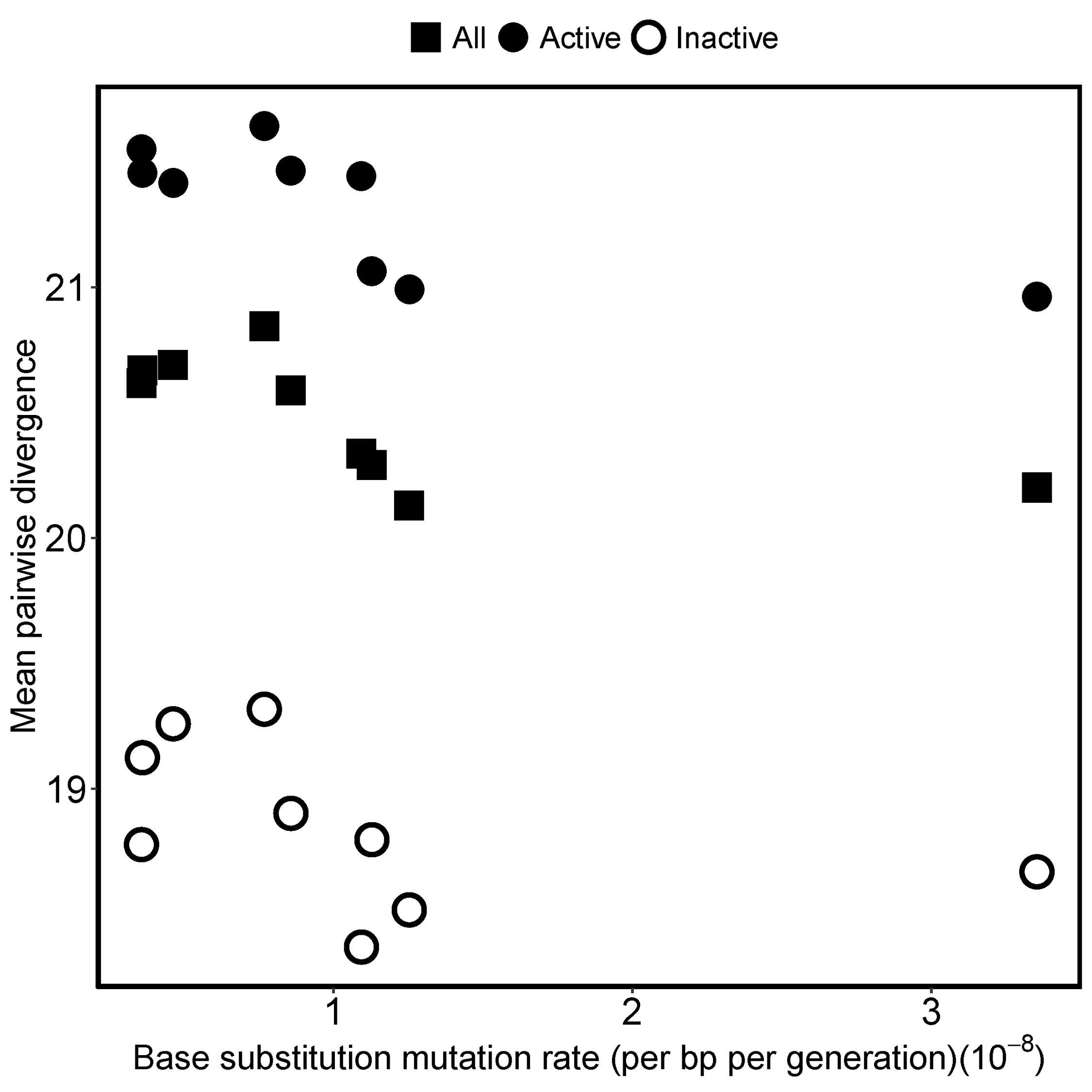

Supplement: S16 Fig — Divergence averaged across all TE families, only active TE families, and only inactive TE families are plotted as filles squares, filled circles, and empty circles, respectively. (TIF) [file pgen.1009827.s048.tif]

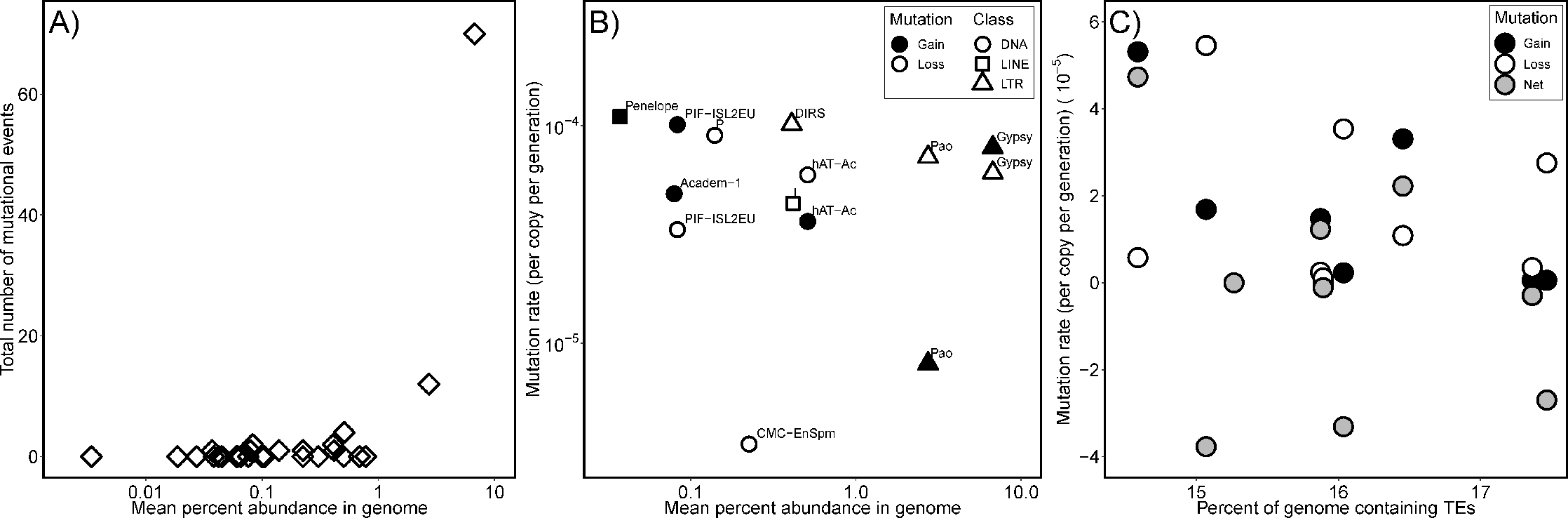

Supplement: S17 Fig — Percent abundance (log scale) of each TE family averaged across genotypes in D. magna plotted against (A) number of mutation events for each TE family and (B) gain and loss rates for each active TE family. Gain rates for DNA/CMC-EnSpm, DNA/P, LTR/DIRS, LINE/I and loss rates for DNA/Academ-1, LINE/Penelope are not shown because there were zero mutation events. (C) Percent of the genome occupied by TEs for each assembly plotted against the TE gain (black), loss (white) and net (grey) rates averaged across all families and MA lines. Percent abundance of TEs was estimated using the read mapping approach. (TIF) [file pgen.1009827.s049.tif]
