# Supplementary material for: Engines of change: Transposable element mutation rates are high and variable within Daphnia magna
Source: PLoS Genet. 2021 Nov 1;17(11):e1009827. doi: 10.1371/journal.pgen.1009827 (PMC8594854; doi:10.1371/journal.pgen.1009827)
Supplement: Attachment — Submitted filename: 090921_CoverLetter_RtR.pdf [file pgen.1009827.s050.pdf]

SARAH SCHAACK, PhD
Reed College
Biology Department
3203 SE Woodstock Boulevard, Portland, Oregon 97202-8199
phone: 503-517-7948 fax: 503-777-7239

September 9, 2021

Dr. Gregory S. Barsh
Editor in Chief, PLoS Genetics

Dr. Lindi Wahl
Associate Editor

Dr. Kirsten Bomblies
Section Editor

Dear Editors,

We are submitting our revised manuscript, **Engines of change: Transposable element mutation rates are high and variable within populations of *Daphnia magna***. The comments from the three reviewer were extremely helpful in improving the readability and clarity of our study, and we thank them, and you, for your time and careful consideration of our study.

Please find a point-by-point response to all comments from the reviewers below, and a revised version of the main text, supplemental text, and supplemental tables have been uploaded.

Thank you,

Sarah Schaack, PhD

Comments to the Authors:

Reviewer #1: In this manuscript, Ho, Bellis, et al. investigate transposable element variation and mutation rates in different genotypes of Daphnia magna. The authors explain the different ways TE copy number may change in Daphnia, which is useful to the reader because of the unique aspect of asexuality. The authors find that mutation rates are variable between lines, and interestingly several lines show a directional bias. They also propagated populations of the same genotypes where selection occurred and show that transposable element mobilization is constrained. Overall, the manuscript's data support the authors' conclusions which represent a strong contribution to the field. However, the manuscript could be improved in clarity and organization. There are also a few details missing from the manuscript, and discrepancies, which need to be clarified for full confidence in the paper's findings and conclusions.

We appreciate the reviewers' excellent comments and suggestions, and have responded point-by-point, below.

1. The results section was challenging to read as many results were listed and it was difficult to extract take-home messages. I suggest further subdividing the results section, and making subsection titles more informative and summarizing the results. For example, the authors may consider subheadings similar to these:

Line 167:
Characterizing TE content in Daphnia
Line 187:
Variation in TE activity on a long-term scale
Line 214:
Estimated rates of TE loss and gain using mutation accumulation lines
Line 236:
TE activity is under selective constraint
Line 245:
Validation of methods for detecting TE insertion mutations
Line 259:
TE mutation rates are not correlated with other types of mutation

This is a great suggestion, and we added the subheadings (in all but one case, and in that case --since it would have been a heading for a single paragraph-- we simply reworded the first sentence of the paragraph to emphasize the main finding more clearly, is it related to the larger section in which it was housed).

2. The authors use simulations to estimate false positive and false negative rates. They mention in the discussion that read depth can alter the false positive and negative rates, however it seems this was not factored into the simulations. Please clarify if there was a correlation between depth and number of insertions detected.

This is an excellent point, so we tested for it (see below and Table S18C), and also added a Supplemental Table (S18B) with our median read counts for each lineage, and described the results in the Supplemental Results (Lines 291-293).

Within genotypes where TE insertions were discovered in MA or EC lines (FA, FC, GA, GB, GC, IB, IC). We did not find a significant Pearson's correlation between the median depth of coverage in the genome and the number of TE insertions. The median depth of coverage for all lines are now listed in Table S18B, and we note the lack of correlation (illustrated below) in the Supplemental Results.

**Genotype**	**Correlation**	**t**	**df**	**P-value**
FA	-0.228	-0.620	7	0.555
FC	-0.492	-1.598	8	0.149
GA	0.069	0.195	8	0.850
GB	-0.580	-2.014	8	0.079
GC	-0.400	-1.234	8	0.252
IB	0.193	0.557	8	0.593
IC	-0.213	-0.617	8	0.554

We agree with the reviewer that read depth can alter the likelihood of false positive and false negative calls. Chen and Zhang (2021; https://doi.org/10.1093/molbev/msab073) showed that there was a correlation between read depth and TE detection when depth was below 20x, but not when depth was above 20x. Given the lack of correlations and that all our lines had a median depth of coverage above 20x (Table S18B), we do not believe that the differences in depth had a significant effect on the detection of TE mutations in our experiment.

Why was 50x depth used for the simulations and not the empirical depth, which will vary between different lines? I could not find any mention of the empirical depth in the manuscript or supplement.

We now provide the empirical median depth of coverage for each line on Table S18B. We utilized 50x depth in the simulations because the empirical median depth of coverage was approximately 50 when averaged across all lines (Table S18B). We now state this in our Supplemental Methods (Line 151-153 in the Supplement).

We agree that the simulations do not perfectly match the variation in depth found across our lines. However, as mentioned above, the depth for our lines are well above the threshold (20x) where Chen

and Zhang (2021) found an effect of depth on TE detection. Thus, we do not believe that matching the empirical variation in depth into our simulations would greatly affect our estimates of FDR and FOR.

3. Confidence intervals are overlapping for TE losses between ECs and MA lines in Table 2. Why are the bootstrap results presented when a mixed effect model was referred to in the main text? Why is the mixed effect model more suitable than the bootstrap, as the two methods result in a discrepancy in significance for TE loss rates?

The bootstrapped 95% confidence intervals (CI) of the mean for TE loss rates do not actually overlap between the MA (0.53 to 3.23) and the EC lines (0.064 to 0.46). The CIs were used to illustrate the confidence in the estimates of the means, rather than for testing statistical significance (although, even if the CIs overlapped, that in itself would not determine whether rates were significantly different between the treatments). To actually test for statistical differences, we used the generalized linear mixed effect model described on Lines 240-247.

4. Please include a clarification of a couple details about the EC lines. How was the number of generations estimated and what is the confidence of this estimate (e.g. there may be overlapping generations)? Please briefly explain the limitations of your sampling approach of the EC lines, as you may not be capturing variation within the population.

We added a section with these details to the Supplemental Materials and Methods (Lines 2-19 in the Supplement).

5. L251 – please clarify that you are referring to the empirical data, and FDR/FOR didn't greatly vary between the mutation types

We have modified this section to clarify this (Line 254-256).

6. Why were the EC rates not adjusted in the table S9? Please include explanation in the simulations section and/or the table legend.

We have now adjusted the rates for EC lines as well on Table S9.

7. L281-283: Each unknown element identified by RepeatModeler may represent a family, and in theory could have a family-specific mutation rate. I think you should say class or superfamily -level specific rates.

Corrected on Line 285.

8. Please try to reword the sentence on lines 401-403 to increase clarity.

Reworded on Lines 403-406.

9. In comparison of D. pulex and D. magna TE content, why is the RepeatMasker method used when you state it is a poor method for comparing different assemblies especially those of different qualities?

The RepeatMasker method is the most commonly used method in the literature, and so for cross-species and cross-study comparisons, it seemed wise to keep the methodology consistent. In addition, on Table S18A, we show that our *D. magna* assemblies and the *D. pulex* assembly are fairly comparable, with similar N50 values and gene content (based on BUSCO analysis using the Arthropod reference gene set). We have added this information in the Results (Lines 162-165, 501-504).

Reviewer #2: This is a very well-written article that sheds light not only on the abundance and diversity of transposable elements (TEs) in populations of Daphnia magna across a latitudinal gradient and how this compares with that of the closely related species D. pulex, but also on estimating mutation rates with and without selection across populations. Determining whether transposition rates vary across populations is a fundamental and still open question in the field, and the results presented in this manuscript do contribute to answer it.

I have a few suggestions for the authors to consider.

We thank the reviewer for the positive feedback and helpful suggestions. Please find our corrections and additions in response to each point below.

One of the metrics used to estimate overall abundance is affected by the quality of the assemblies, as mentioned by the authors. The authors apparently used previously available assemblies. Would the authors consider adding a few lines about the quality of the assemblies? Is it comparable across genomes? Are these assemblies based on long-reads? how the variation in assembly impacts the abundance estimates? Part of this analysis is currently in supplemental material, I think it deserves a brief mention in the results section as well. Along the same lines, how does the assemblies of D. magna and D. pulex compare?

Our *D. magna* assemblies are based on short read data, using a reference genome as a guide (see Supplemental Methods). On Table S18A, we show assembly statistics and results from BUSCO analyses to evaluate the quality of the *D. magna* and *D. pulex* assemblies. These results suggest that the assemblies are all relatively good quality and are also fairly similar in quality, meaning the estimates of TE abundance between genotypes and species are likely comparable.

We have added this information in the Results in the main text (Lines 162-165) and Methods section (Lines 487, 501-504).

Line 175. Maybe mention what does “t8” stand for?

That is the t-statistic, but includes the subscript to note the degrees of freedom.

Line 205 Do you mean “higher MPD in TE families that were currently active”?

Yes! Thank you for catching that confusing typo.

Line 482. Was redundancy not remove from the TE library? How can this affect the annotations?

Redundancy was removed from the TE library using cd-hit-est. We now state this in the Methods (Lines 495-497).

Line 529. I would encourage the authors to upload their TE library to a public repository such as Dfam so that it is more easily accessible to potential users rather than providing (or additional to providing it) as a supplemental data file.

This is a great suggestion, we have initiated the process to obtain a Dfam account, however these are reviewed manually and it may take some time. We will upload the TE library to this database, in addition to making it available upon request. If our article is accepted, the documentation on the availability of the file will certainly be added to the final version.

Figure 1. Why losses from 1 to 0 cannot be due to cut and paste transposition?

They can, certainly (that possibility is included in the top line "Transposition/Retrotransposition"), but we are also trying to make sure the readers know that there are a number of other mechanisms that can be at play.

Figure 2. Increase contrast of the map so that sampling collections are more conspicuous.

Done.

Figure 3. Are the gain and loss rates of FB zero? Maybe mention it in the legend if that is the case. Same for figure 5A, FB and IB.

We have added a thicker line for FB and IB in Figure 3A and 5A to show that their rates are zero. We also indicated the genotypes with zero rates in the captions of these figures.

Typos in lines 115, 550

All fixed, thank you.

Reviewer #3: This paper quantifies the transposable element activity in a 30-month mutation-accumulation experiment involving 9 genotypes of Daphnia magna. The results are compared to large populations of the same genotypes, in which selection is allowed to act. A total of 95 mutation events are recorded in the MA lines, 70 of which involved gypsy-elements. The mutation rates vary widely between lines and are much lower in the large control populations. I find this an interesting and well-presented study.

Thank you very much for the feedback and suggestions, they have all been implemented and we have added a reference.

I have only minor comments:

line 65-68. Inducing structural variation in the genome might be added to this list.

Done, and we provided an example citation from Kou et al. 2020.

line 196-199. I am not sure I see this distinction (you use "or"). Both types of processes operate at the same time, I would think.

We agree, and made the change to 'and'.

line 205. I think this should be “higher”. Lower is what you expect.

Corrected, thank you.

line 302. Chen and Zhang reanalysed Bast et al. 2019, not 2016.

Corrected in two places, thank you again!

line 404-405. This is an important point, worth emphasising. It extends to TE family differences.

Emphasized on Line 404.

line 418-422. Perhaps selection is acting on DNA repair mechanisms?

Excellent point, we added it to Line 425-426.

line 427-428. I find it difficult to get a sense of how the Daphnia TE mutation rates compare to these other organisms, except Drosophila, which is mentioned on line 347).

This is an excellent point. It is actually a bit of a challenge to compare rate estimates among species at this point, because a) in most cases, only 1 family is assayed, b) a different or know denominator is provided for the rate, or c) differences in methodology (e.g., southern blot versus sequencing) make for apples to oranges comparisons. We are writing a review paper on this issue currently, where we include a massive table (n > 100 studies) with all current empirical estimates of rates, however we are reticent to make any biological inferences from those rates since their genesis is not uniform and makes comparisons of difference potentially very artifactual. Basically, we think the verdict is still out on whether the rates, even among invertebrates, are going to be highly variable or similar across taxa, and think this is an area in need of further investigation.

line 477. Please mention briefly the sequencing technique (Illumina short reads) and assembly method (reference-guided) here.

Added.

line 512. Sentence truncated.

This sentence is removed now.

Have all data underlying the figures and results presented in the manuscript been provided?

Large-scale datasets should be made available via a public repository as described in the *PLOS Genetics* data availability policy, and numerical data that underlies graphs or summary statistics should be provided in spreadsheet form as supporting information.

Reviewer #1: **No:** Code is accessible on the Github page, however I was unable to access data at the accession PRJNA658680 at NCBI. Please ensure the sequencing data is publicly available before full acceptance.

The accession PRJNA658680 is now available on NCBI.

Reviewer #2: **No:** Some will be provided upon acceptance

Reviewer #3: Yes
